# Quorum sensing regulates heteroresistance in *Pseudomonas aeruginosa*

**DOI:** 10.3389/fmicb.2022.1017707

**Published:** 2022-10-28

**Authors:** Yang Lu, Yuyang Liu, Chenxu Zhou, Yaqin Liu, Yifei Long, Dongling Lin, Rui Xiong, Qian Xiao, Bin Huang, Cha Chen

**Affiliations:** ^1^Department of Laboratory Medicine, The Sixth Affiliated Hospital of Guangzhou Medical University, Qingyuan People’s Hospital, Qingyuan, China; ^2^Department of Laboratory Medicine, The Second Affiliated Hospital of Guangzhou University of Chinese Medicine, Guangzhou, China; ^3^Department of Laboratory Medicine, Chengdu First People's Hospital, Chengdu, China; ^4^Department of Laboratory Medicine, The First Affiliated Hospital of Sun Yat-sen University, Guangzhou, China; ^5^The Second Clinical College of Guangzhou University of Chinese Medicine, Guangzhou, China

**Keywords:** quorum sensing, heteroresistance, *Pseudomonas aeruginosa*, oprD, efflux pumps gene, fitness cost

## Abstract

The prevalence and genetic mechanism of antibiotic heteroresistance (HR) have attracted significant research attention recently. However, non-genetic mechanism of HR has not been adequately explored. The present study aimed to evaluate the role of quorum sensing (QS), an important mechanism of behavioral coordination in different subpopulations and consequent heteroresistance. First, the prevalence of HR to 7 antibiotics was investigated in 170 clinical isolates of *P. aeruginosa* using population analysis profiles. The results showed that *P. aeruginosa* was significantly heteroresistant to meropenem (MEM), amikacin (AMK), ciprofloxacin (CIP), and ceftazidime (CAZ). The observed HR was correlated with down-regulation of QS associated genes lasI and rhlI. Further, loss-of-function analysis results showed that reduced expression of *lasI* and *rhlI* enhanced HR of *P. aeruginosa* to MEM, AMK, CIP, and CAZ. Conversely, overexpression of these genes or treatment with 3-oxo-C12-HSL/C4-HSL lowered HR of *P. aeruginosa* to the four antibiotics. Additionally, although downregulation of *oprD* and upregulation of efflux-associated genes was evident in heteroresistant subpopulations, their expression was not regulated by LasI and RhlI. Moreover, fitness cost measurements disclosed higher growth rates of PAO1Δ*lasI* and PAO1Δ*rhlI* in the presence of sub-MIC antibiotic as compared with that of wild-type PAO1. Our data suggest that under temporary antibiotic pressure, downregulation of QS might result in less fitness cost and promote HR of *P. aeruginosa*.

## Introduction

Heteroresistance (HR) is a phenomenon whereby subpopulations of seemingly isogenic bacteria exhibit different susceptibilities to a particular antibiotic (El-Halfawy and Valvano, 2015). Previous studies have demonstrated the prevalence of heteroresistance in clinical pathogens such as *Klebsiella pneumonia* (Nimmo et al., 2020), *Streptococcus pneumonia* (Engel et al., 2014), *Mycobacterium tuberculosis* (Nimmo et al., 2020), *Staphylococcus aureus* (Nurjadi et al., 2021), *Acinetobacter baumannii* (Charretier et al., 2018; Rodriguez et al., 2019), *Pseudomonas aeruginosa* (Lin et al., 2019; Choby et al., 2021), and *Helicobacter pylori* (Sun et al., 2018). A heteroresistant subpopulation of bacteria can rapidly replicate in the presence of an antibiotic where other susceptible bacteria cells are killed (Band and Weiss, 2019). This ultimately leads to failure of antibiotic treatment and hence induce bacterial resistance to drugs.

Currently, research on the mechanism of bacterial HR mainly focuses on point mutations. Amplification of chromatin fragments in Gram-negative bacteria has been found to be the prevalent mechanism for balancing resistance and the fitness costs of resistance mutation in the presence of antibiotics by spontaneous tandem amplifications rather than by heritable mutations (Nicoloff et al., 2019). Besides, bacteria can generate subpopulations at a high fitness cost of resistance through point mutations under the selection pressure of antibiotic exposure or compensatory secondary mutations in the absence of antibiotic. Therefore, this reduces the fitness cost and possibly restores the resistance of the native populations (Paradise et al., 1998; Lee et al., 2011). HR may be a strategy for bacteria to adapt to the environment under the antibiotic selection pressure before resistance is acquired. However, it is not yet clear whether a non-genetic mechanism could generate less fitness cost and consequently induce heteroresistance during the short-term evolutionary course. This study evaluated the quorum sensing (QS) system, which is the most common mechanism for coordination of activities in bacterial cells and population-density changes in behavior, such as HR.

*Pseudomonas aeruginosa* is one of the main causes of nosocomial infections. It is associated with extremely high mortality and morbidity in patients with cystic fibrosis and immunocompromised individuals (Sadikot et al., 2005; Chen et al., 2019). Furthermore, *P. aeruginosa*, a model bacterium for studying QS systems, has two main QS systems, *las* and *rhl*, which are responsible for synthesis of the N-acyl homoserine lactone signal molecules, N-(3-oxododecanoyl)-L-homoserine lactone (3-oxo-C12-HSL), and N-butanoyl-L-homoserine lactone (C4-HSL), respectively (Smith and Iglewski, 2003; Chen et al., 2019b). The 3-oxo-C12-HSL and C4-HSL bind to and activate cognate transcription factors, LasR or RhlR, respectively, and induce formation of biofilm and expression of various virulence factors (Moura-Alves et al., 2019). However, resistant *P. aeruginosa* strains have evolved due to the abuse of antibiotics such as aminoglycosides, quinolones, and β-lactams (De Oliveira et al., 2020). Recently, studies have shown HR in *P. aeruginosa* to carbapenems (Mei et al., 2015; He et al., 2018), cefepime (Jia et al., 2020), and colistin (Lin et al., 2019). HR may be correlated with upregulation of efflux-associated genes and downregulation of the porin-encoding gene *oprD* in resistant subpopulations (Ikonomidis et al., 2008). However, the prevalence and mechanism underlying HR are still not clear. Therefore, the current study extensively investigated the mechanism of HR to provide new insights into clinical treatment efficacy for bacterial infections and *P. aeruginosa* resistance.

The present study focused on the role of QS system in HR of *P. aeruginosa*. The prevalence of HR to several antibiotics in clinical *P. aeruginosa* isolates was characterized, which showed that downregulation of LasI/RhlI was associated with increased HR. Further, it was evident that restoration of LasI/RhlI transcription significantly suppressed HR in *P. aeruginosa*. Notably, the current study showed that fitness cost, but not *oprD* and efflux-associated genes, was involved in LasI/RhlI-inhibited HR. These findings may provide new and significant insights into regulatory networks of HR, and offer some novel targets for prevention of antibiotic resistance.

## Materials and methods

### Bacterial strains

A total of 170 non-duplicate clinical *P. aeruginosa* isolates were obtained from two tertiary hospitals in Guangzhou between 2017 and 2018. The isolates were stored at −80°C and cultured on blood agar plate before the experiments were conducted. All isolates were reconfirmed using matrix-assisted laser desorption ionization-time of flight mass spectrometry. All bacterial strains and plasmids used in the current study are listed in Supplementary Table 1. The PAO1Δ*lasI* and PAO1Δ*rhlI* strains had been constructed in our previous study (Zeng et al., 2016; Lu et al., 2017). Briefly, the sacB-based suicide vector system was adapted for knockout of *lasI* or *rhlI* in PAO1 bacterial strains.

### Plasmid construction

The pROp200 was used to construct plasmid that expressed LasI or RhlI. The coding sequences of LasI or RhlI were cloned into the EcoRΙ site of pROp200 with a Ready-to-Use Seamless Cloning Kit (BBI, United States, Code NO. B632219) to generate expression vectors named pROp200-*lasI* or pROp200-*rhlI*.

### Antimicrobial susceptibility testing

Meropenem (APE-BIO, United States), Amikacin (BBI Life Science, United States), Ciprofloxacin (BBI Life Science, United States), Ceftazidime (Sigma-Aldrich, United States), Fosfomycin (APE-BIO, United States), polymyxin B (BBI Life Science, United States), and Gentamicin (BBI Life Science, United States) were freshly prepared for each experiment and filter sterilized using a 0.22 μm filter. Mueller-Hinton broth (Oxoid, United Kingdom) supplemented with calcium and magnesium (25.0 mg/l Ca2+ and 12.5 mg/l Mg2+) and Mueller-Hinton II agar (Oxoid, United Kingdom) were used for susceptibility testing *in vitro*. The antibiotic breakpoints for MEM, AMK, CIP, and CAZ were defined by CLSI-M100-S26. Quality control in the experiments was monitored with *P. aeruginosa* strain ATCC 27853 and pandrug-resistant 132A5.

### Fitness cost measurements

Growth rates were analyzed using the biotek (synergy H1) as previously described (Nicoloff et al., 2019). Four colonies of each test strain were used to inoculate four biological replicates in 3 ml of Mueller-Hinton broth. Overnight cultures were diluted 1:1000 with 3 ml of fresh Mueller-Hinton broth, and then allowed to grow for 24 or 48 h at 37°C. Absorbance was measured at 600 nm (OD600) every 2 h, with cultures shaken between measures.

### Population analysis profiles (PAPs)

Heteroresistance of clinical *P. aeruginosa* isolates and PAO1 was determined using PAPs tests according to a previously published protocol with several modifications (Band et al., 2016). Three colonies were used to start three independent overnight cultures in 3 ml Mueller-Hinton broth. Twenty microliters of gradient dilutions of an overnight culture (10^1^, 10^2^, 10^3^, 10^4^, 10^5^, 10^6^, and 10^7^ CFU/ml) were plated on Mueller-Hinton agar plates supplemented with or without increasing amounts (2-fold increments) of Amikacin (AMK, 1–64 mg/l), Meropenem (MEM, 0.5–16 mg/l), Ciprofloxacin (CIP, 0.25–8 mg/l), Ceftazidime (CAZ, 1–32 mg/l), Fosfomyci (FB, 0.125–8 mg/l), PolymyxinB (PB, 0.125–8 mg/l) or Gentamacin (GM, 0.125–8 mg/l). Plates were incubated overnight at 37°C and colonies were eventually counted and recorded. Heteroresistance was defined as the presence of a subpopulation of cells capable of growing at antibiotic concentrations at least eight-fold higher than the highest concentration that does not affect the replication of the dominant population. Quality control was monitored using *P. aeruginosa* strain ATCC 27853 and 132A5 (clinical pandrug-resistant strain isolated in our laboratory).

### Stability of antibiotic resistance and heteroresistance

Two clones isolated from plates with the highest drug concentration were supplemented with 3 ml of Mueller-Hinton broth and subjected to the same selection pressure overnight. The minimal inhibitory concentrations (MICs) were determined using the broth microdilution method. The cultures were then grown for two more nights. Their MICs after 40 generations in the absence of selective pressure were determined and the resistance was deemed unstable if the MIC clearly decreased or reverted to that of the original parental isolate in at least one of the cultures.

### Genes expression analysis

Cultures of original parental strains and heteroresistance subpopulations were grown in CAMHB medium with or without selection pressure at 37°C with shaking to an OD600 of 0.5. Total RNA of each strain was extracted by RNAiso Plus reagent (TaKaRa, Dalian, Liaoning, China). For qPCR analysis, 20 ng of total RNA was subjected to DNase I digestion PrimeScript RT reagent kit (TaKaRa, Dalian, Liaoning, China) at 37°C for 30 min and then to heat inactivation of DNase I at 65°C for 10 min. The cDNA was subjected to quantitative PCR (qPCR) on a ViiATM 7 Dx system (Applied Biosystems, Foster, CA, United States) using the SYBR green qPCR master mixes (TaKaRa, Dalian, Liaoning, China). The temperature cycle profile for the qPCR reactions was 95°C for 30s, and then 40 cycles of 95°C for 5 s, 60°C for 20s, and 72°C for 10s. To verify the specificity of PCR product, melting curve analysis was performed immediately after amplification, as follows: heating to 95°C for 20s, cooling to 60°C for 20s, followed by a temperature increase to 95°C with a transition rate of 0.11°C/s while continuously collecting the fluorescent signal. The transcript levels of the target genes were normalized to the expression of an internal control gene (*rpoD*) using the 2^-ΔΔCt^ method. All reactions were run in triplicate. The cycle threshold (Ct) values should not differ more than 0.5 among triplicates. Sequences of the primers used in the current study are as listed in Supplementary Table 2.

### Pyocyanin production assay

The detection of pyocyanin, rhamnolipid and biofilm formation was performed as described in our previous study (Lu et al., 2019). Three clones isolated from PAPs test plates with 0, 2, 4, 8 or 16 mg/l of AMK were supplemented with 3 ml of Mueller-Hinton broth and subjected to the same selection pressure overnight. Overnight cultures were diluted 1:100 with 10 ml of fresh Mueller-Hinton broth and subjected to the same selection pressure, then allowed to grow for 24 h at 37°C. Pyocyanin in 5 ml of *P. aeruginosa* culture supernatant was extracted with 3 ml of chloroform and 1 ml of 0.2 N HCl, and then the absorbance of the extract was measured at 520 and 600 nm. The concentration of pyocyanin was determined using the following formula: (A520/A600 × 17.072) = μg/ml (Essar et al., 1990). All of the tests were performed independently and at least in triplicate.

### Rhamnolipid assay

Three clones isolated from PAPs test plates with 0, 2, 4, 8 or 16 mg/l of AMK were supplemented with 3 ml of Mueller-Hinton broth and subjected to the same selection pressure overnight. Overnight cultures were diluted 1:100 with 10 ml of fresh M9 (containing 0.4% glucose, 2 mM MgSO4 and 100 μM CaCl_2_) and subjected to the same selection pressure, then allowed to grow for 20 h at 37°C. Next, 1 ml of culture supernatant was acidified (pH 2.5 ± 0.2) using 1 N HCl, rhamnolipid was extracted with 4 ml chloroform. Then 3 ml of chloroform extract was allowed to react with 100 μl of 1 g/l of methylene blue and 2 ml of distilled water, the OD638 was measured (Pinzon and Ju, 2009). All results were analyzed of three independent experiments.

### Biofilm formation assay

Three clones isolated from PAPs test plates with 0, 2, 4, 8 or 16 mg/l of AMK were supplemented with 3 ml of Mueller-Hinton broth and subjected to the same selection pressure overnight. Overnight cultures were diluted 1:100 with 10 ml of fresh Mueller-Hinton broth, 20 ml of the dilutions were plated into 96-well culture and subjected to the same selection pressure, then allowed to grow for 24 h at 37°C. Biofilm cells attached to plates were stained with 1% crystal violet. Then, 2 ml of 95% ethanol was used to solubilize crystal violet-stained biofilm cells and measured at OD600 (Carloni et al., 2017). All analyses included three independent experiments.

### Statistical analysis

Data are expressed as the means ± standard errors of the means (SEMs) from at least three independent experiments. The differences between groups were analyzed using the Student’s t-test when two groups were compared or a one-way analysis of variance (ANOVA) when more than two groups were compared. All analyses were performed using GraphPad Prism, version 5 (GraphPad Software, Inc., San Diego, CA, United States). All statistical tests were two-sided; *p* values of <0.05 were considered statistically significant.

## Results

### Prevalence of HR in Clinical *Pseudomonas aeruginosa* isolates

**Figure 1:**
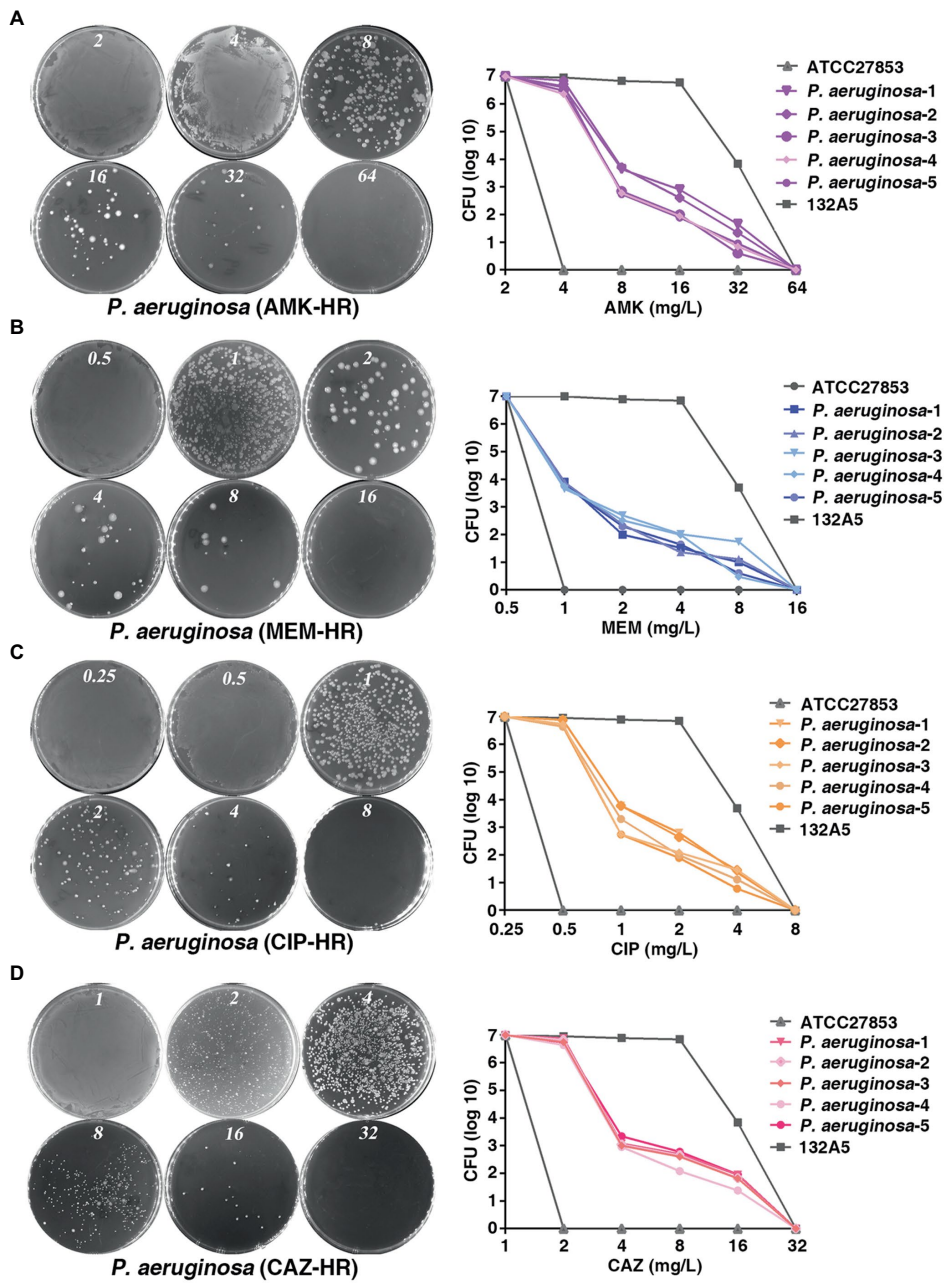
Prevalence of heteroresistance in clinical *Pseudomonas aeruginosa* isolates. **(A–D)** *P. aeruginosa* was heteroresistant to AMK, MEM, CIP and CAZ, respectively. PAPs were performed in five clones of *P. aeruginosa* isolate. Both the sensitive (ATCC 27853) and resistant strains (132A5) of *P. aeruginosa* were used as control. AMK, amikacin; MEM, meropenem; CIP, ciprofloxacin; CAZ, ceftazidime.

To investigate the prevalence of HR to different antibiotics covering major classes in the clinical *P. aeruginosa* isolates, a total of 170 strains were randomly selected and PAPs was performed based on the HR definition by Herve and Karin (Nicoloff et al., 2019). They defined HR as the presence of a subpopulation of cells capable of growing at antibiotic concentrations at least eight-fold higher than the highest concentration that does not affect the replication of the dominant population and resistant subpopulations at frequencies of 1 × 10^−7^ or higher. Of the 170 isolates, 113 (66.5%), 99 (58.2%), 87 (51.2%) and 69 (34.1%) were heteroresistant to amikacin (AMK; Figure 1A), meropenem (MEM; Figure 1B), ciprofloxacin (CIP; Figure 1C) and to ceftazidime (CAZ; Figure 1D), respectively. Notably, 55 isolates (32.4%) showed a cross-HR to all the four antibiotics (AMK, MEM, CIP, and CAZ). In conclusion, a high frequency of HR to AMK, MEM, CIP, and CAZ was observed in different clinical isolates of *P. aeruginosa* (Table 1). However, HR with fosfomyci (FB), PolymyxinB (PB) and Gentamacin (GM) was not observed in the present investigation. In addition, the current study also assayed the heteroresistance of PAO1, a strain most commonly used in laboratory research. Results of the present study showed that PAO1 was also heteroresistant to MEM, AMK, CIP, and CAZ (Supplementary Figure 1).

**Table 1:** Heteroresistance (HR) of clinical *Pseudomonas aeruginosa* isolates(*n* = 170).

Antibiotics	Resistance	Resistance Ratio(%)	HR	HR Ratio(%)
AMK	37	21.8	113	66.5
MEM	50	29.4	99	58.2
CIP	44	25.9	87	51.2
CAZ	15	8.8	58	34.1
FOS	10	5.8	0	0
PB	15	8.8	0	0
GM	12	7.0	0	0

### Expression of LasI and RhlI is negatively correlated with HR of *Pseudomonas aeruginosa*

**Figure 2:**
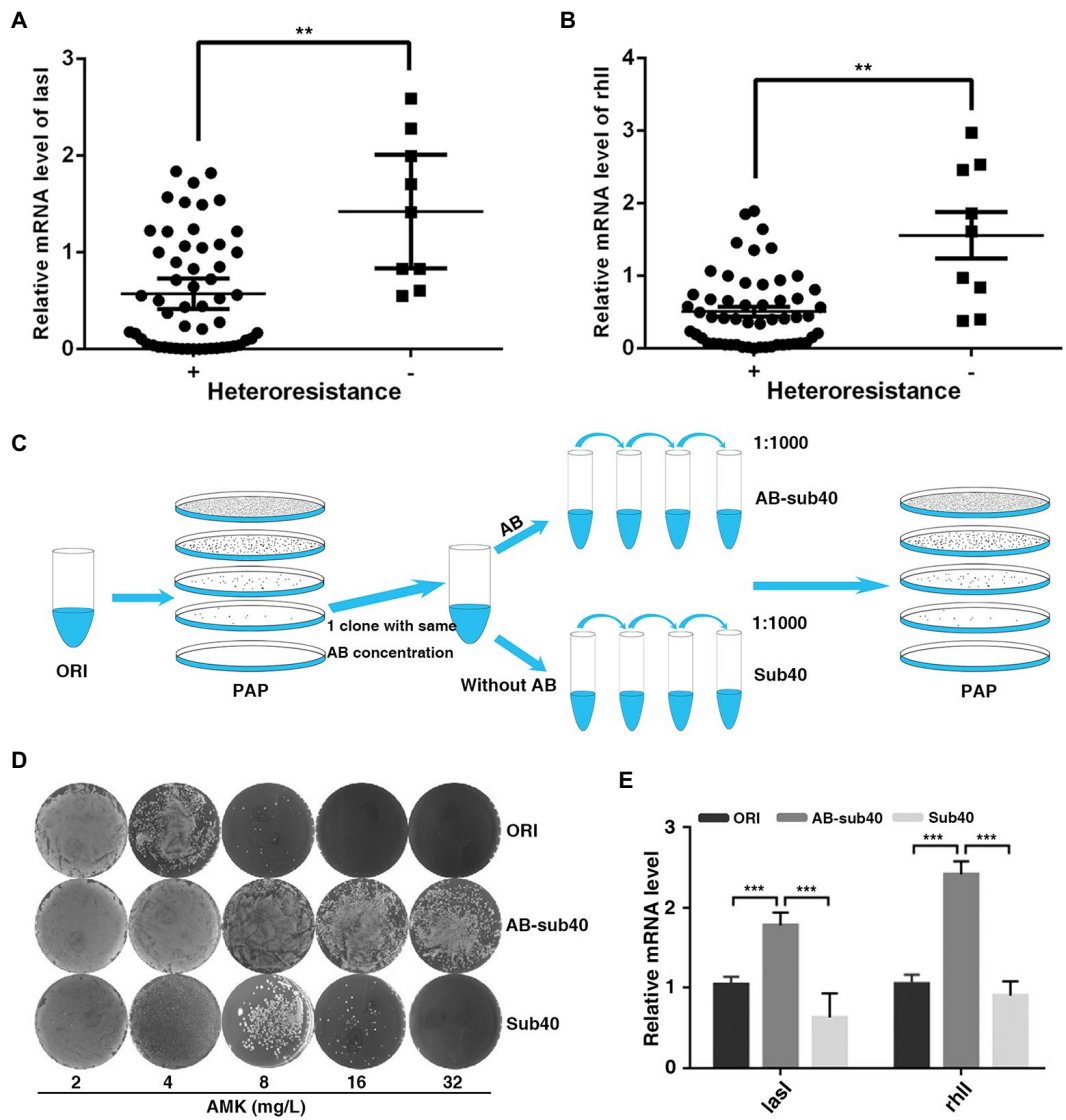
Expression of LasI and RhlI is negatively correlated with the HR of *P. aeruginosa*. **(A,B)** Expression of LasI and RhlI was lower in the heteroresistance-positive group compared with in the heteroresistance-negative group. **(C)** A schematic outline of the methods used to assess the stability of HR. A resistant colony from the PAPs test plates was re-streaked and grown at the same concentration of antibiotics (Enrich), followed by serial passages with or without antibiotic selection for 40 generations (1, 1,000 daily dilution). **(D)** After serial passages in antibiotic-free medium, resistant subpopulations reverted to the heterogeneous resistance phenotype showed by the original population. **(E)** The expression of LasI and RhlI in the original PAO1 (ORI), resistant subpopulations after 40 generations with or without exposure to antibiotics (AB-sub40 and sub40, respectively). **, *p* < 0.01; ***, *p* < 0.001.

To determine the biological significance of QS system in regulation of heteroresistant, the current study analyzed the correlation between LasI/RhlI levels and heteroresistant features. In total, 55 isolates displayed HR to all four antibiotics (MEM, AMK, CIP, and CAZ) were defined as heteroresistant positive, whereas 9 isolates that showed no heteroresistance to any of these antibiotics were defined as heteroresistant negative. The expression of LasI and RhlI and QS-regulated biofilm formation potential in heteroresistant positive group of isolates was lower than that in the negative group (Figures 2A,B; Supplementary Figure 2A). Furthermore, compared with original, expression of LasI and RhlI and QS-regulated rhamnolipid, pyocyanin synthesis and biofilm formation in resistant subpopulations was also down-regulated (Supplementary Figures 2B–E). These results indicated that QS system may be negatively correlated with heteroresistance in *P. aeruginosa*.

Typically, after serial passages in antibiotic-free medium, some highly resistant subpopulations of *P. aeruginosa* revert to the heterogeneous resistance phenotype shown by the original population (Nicoloff et al., 2019). Therefore, the phenotypic stability and expression of LasI and RhlI was assessed in PAO1 in the present study. PAPs analysis was simultaneously performed on the original PAO1 (ORI) and resistant subpopulations (Enrich), after 40 generations with or without exposure to AMK or CIP (AB-sub40 and Sub40 respectively, Figure 2C). Results showed that ORI and Sub40 were heteroresistant positive, whereas the AB-sub40 became to the resistant strain (Figure 2D; Supplementary Figure 3A). This was an indication that resistance of subpopulations (Enrich) would be effectively enhanced by exposure to long-term antibiotics pressure. It was also demonstrated that the resistance of subpopulations (Enrich) was almost restored to the level of native populations after 40 generations without exposure to antibiotics. Furthermore, it was found that the expression of LasI and RhlI in AB-sub40 group was higher than that in the ORI and Sub40 groups (Figure 2E; Supplementary Figure 3B). These results showed that QS system may be negatively correlated with heteroresistance in *P. aeruginosa*.

### LasI and RhlI inhibit HR in *Pseudomonas aeruginosa*

**Figure 3:**
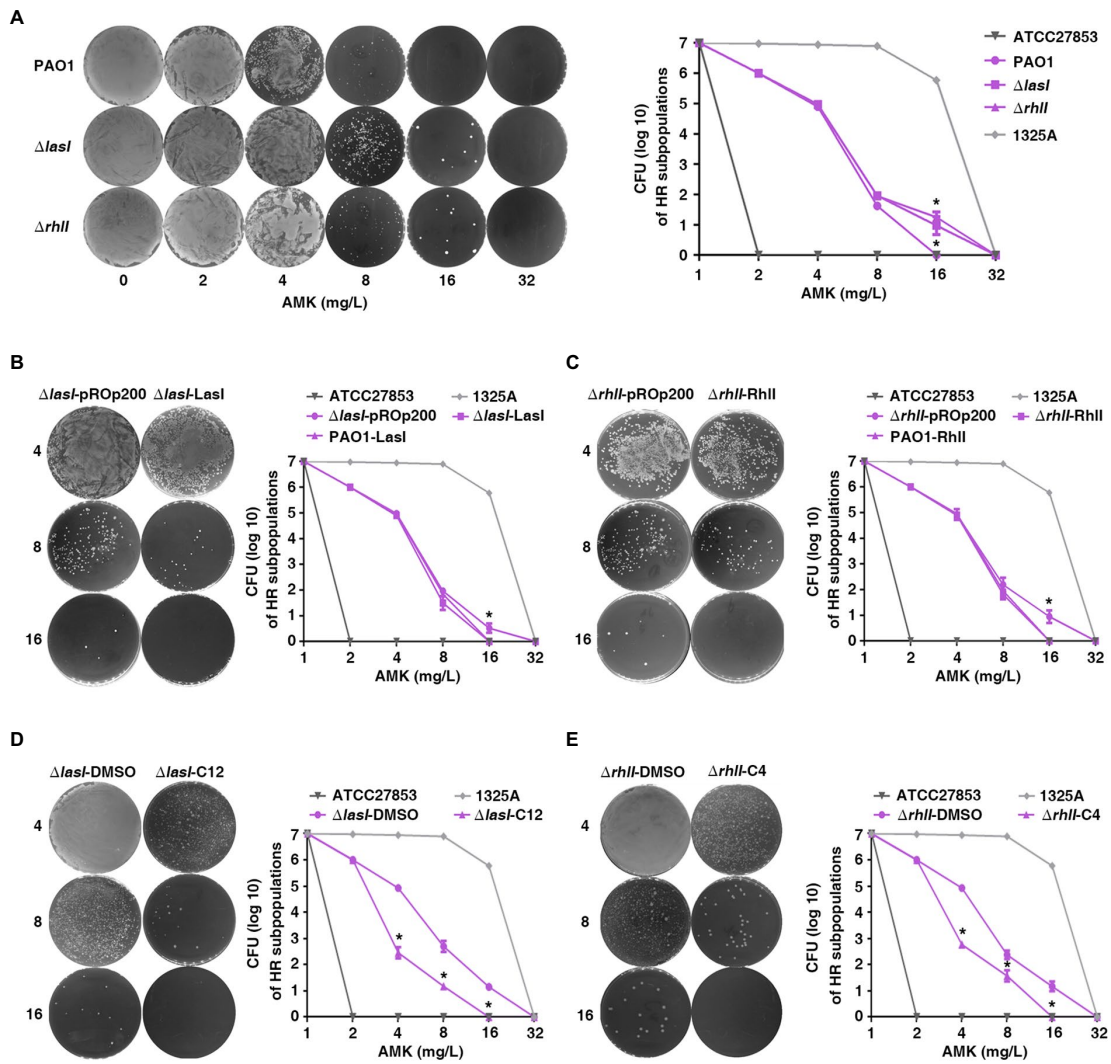
The quorum sensing system of *P. aeruginosa* inhibits heteroresistance. **(A)** Deficiency of *lasI* or *rhlI* in PAO1 enhanced heteroresistance. *, *p* < 0.05, PAO1Δ*lasI* or PAO1Δ*rhlI* compared with PAO1; **(B,C)** Over expression of *lasI* or *rhlI* inhibited heteroresistance. *, *p* < 0.05, Δ*lasI*-LasI compared with Δ*lasI*-pROp200; Δ*rhlI*-RhlI compared with Δ*rhlI*-pROp200. **(D,E)** Exogenous 3-oxo-C12-HSL (C12) or C4-HSL (C4) inhibited heteroresistance. The HR tests (PAPs) were carried out on PAO1Δ*lasI* and PAO1Δ*rhlI* treated with 40 μM of 3-oxo-C12-HSL and C4-HSL, respectively, *, *p* < 0.05, Δ*lasI*-C12 compared with Δ*lasI*-DMSO; Δ*rhlI*-C12 compared with Δ*rhlI*-DMSO. Δ*lasI*-pROp200: PAO1Δ*lasI* strain transformed with pROp200 plasmid; Δ*lasI*-LasI: PAO1Δ*lasI* strain transformed with pROp200 plasmid that expressed LasI; Δ*lasI*-DMSO: PAO1Δ*lasI* strain treated with DMSO; Δ*lasI*-C12: PAO1Δ*lasI* strain treated with 3-oxo-C12-HSL; Δ*rhlI*-DMSO: PAO1Δ*rhlI* strain treated with DMSO; Δ*rhlI*-C4: PAO1Δ*rhlI* strain treated with C4-HSL.

To explore the effect of QS on heteroresistance to antibiotics in *P. aeruginosa*, PAPs was also performed with PAO1 and *lasI* or the *rhlI* single gene-deficient strains (PAO1Δ*lasI* and PAO1Δ*rhlI*). Subpopulations of PAO1Δ*lasI* and PAO1Δ*rhlI* showed an enhanced potential to grow in high concentration of AMK (Figure 3A) compared with the wild-type. This indicated that the deficiency of *lasI* or *rhlI* significantly enhanced heteroresistance of *P. aeruginosa* to AMK. A similar phenomenon was also observed with MEM, CIP, and CAZ antibiotics (Supplementary Figure 4).

To confirm the results of loss-of-function analysis, we performed a gain-of-function analysis using *lasI*-overexpression plasmid or extracellular signal N-(3-oxododecanoyl)-L-HSL (3-oxo-C12-HSL) for PAO1Δ*lasI* and *rhlI*-overexpression plasmid or N-butanoyl-L-homoserine lactone (C4-HSL) for PAO1Δ*rhlI*. Transformation of *lasI*-overexpression plasmid (Δ*lasI-*LasI) or treatment with 3-oxo-C12-HSL (Δ*lasI-*C12) caused significant decrease in subpopulations at 8 and 16 mg/l of AMK for the PAO1Δ*lasI* strain as compared with the control group (Δ*lasI-*pROp200 or Δ*lasI-*DMSO; Figures 3B,D). Further, the overexpression of RhlI or treatment with C4-HSL also impaired the viability of subpopulations for PAO1Δ*rhlI* strain in AMK (Figures 3C,E). In general, the data obtained in the current study showed that QS system may negatively regulate the heteroresistance of *P. aeruginosa*.

### OprD and efflux-associated genes are not involved in QS-inhibited HR in *Pseudomonas aeruginosa*

**Figure 4:**
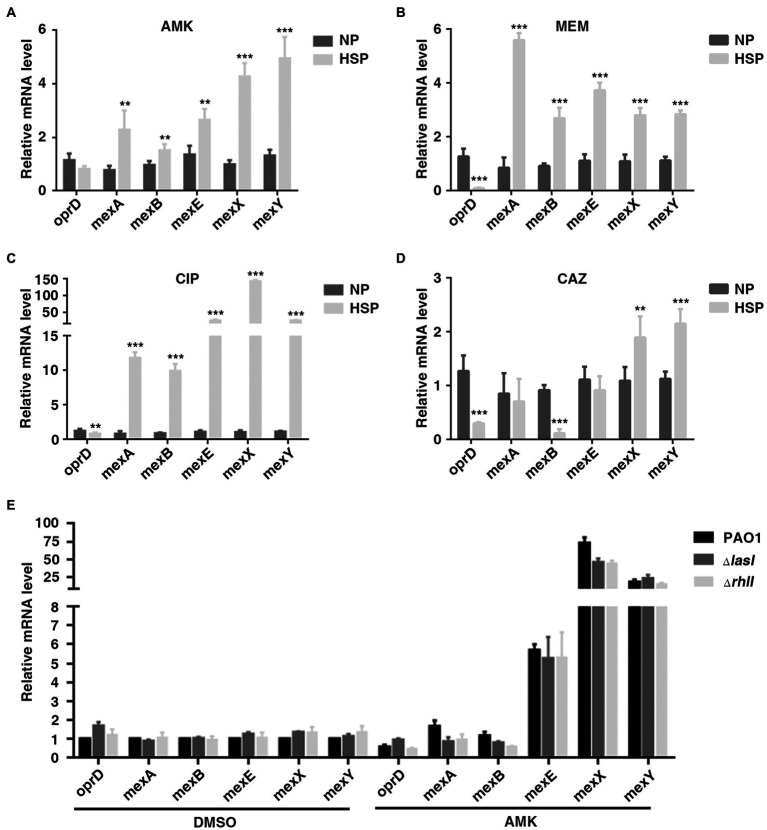
OprD and efflux-associated genes were not involved in QS-inhibited HR. **(A–D)** Expression of OprD and efflux-associated genes in native population (NP) and resistant subpopulation (HSP) of AMK, MEM, CIP, or CAZ. A resistant colony growing on PAPs test plates with highest antibiotic concentration was re-streaked with the same concentration of the indicated antibiotics, followed by real-time PCR analysis. **(E)** The expression of *oprD* and efflux-associated genes was not regulated by LasI and RhlI. PAO1, PAO1Δ*lasI*, and PAO1*ΔrhlI* were cultured in LB for 6 h before qPCR analysis. The *rpoD* gene was used as an internal control. **, *p* < 0.01; ***, *p* < 0.001.

A previous study showed that reduced expression of porin gene *oprD* and increased expression of efflux-associated genes were associated with carbapenem heteroresistance in *P. aeruginosa* (He et al., 2018). The transcription of these genes in PAO1 HR subpopulations (HSP) and native populations (NP) was also tested in different antibiotics. Consistently, the expression of *oprD* was lower in HSP compared with NP for antibiotics AMK, MEM, and CAZ. On the other hand, it was found that the transcription of efflux-associated genes, including *mexA, mexB, mexE, mexX*, and *mexY* increased in HSP for antibiotics AMK, MEM, and CIP (Figures 4A–D). Therefore, the results of the current study demonstrated that both reduced transcription of porin gene *oprD* and increased transcription of efflux-associated genes conferred the subpopulations antibiotic resistance.

The present study also confirmed the involvement of *oprD* and efflux-associated genes in QS system-regulated heteroresistance. It was found that treatment with 2 mg/l of AMK caused downregulation of *oprD* and up-regulation of MexX/MexY. However, compared with wild-type, PAO1Δ*lasI* or PAO1Δ*rhlI* showed no difference in the expression of *oprD* and the efflux-associated genes (Figure 4E). Generally, the obtained data showed that the inhibitory effect of QS system on heteroresistance of *P. aeruginosa* was not mediated by *oprD* and efflux-associated genes.

### Deficiency of *lasI* or *rhlI* favors the survival of PAO1 under sub-MIC concentration of antibiotics

**Figure 5:**
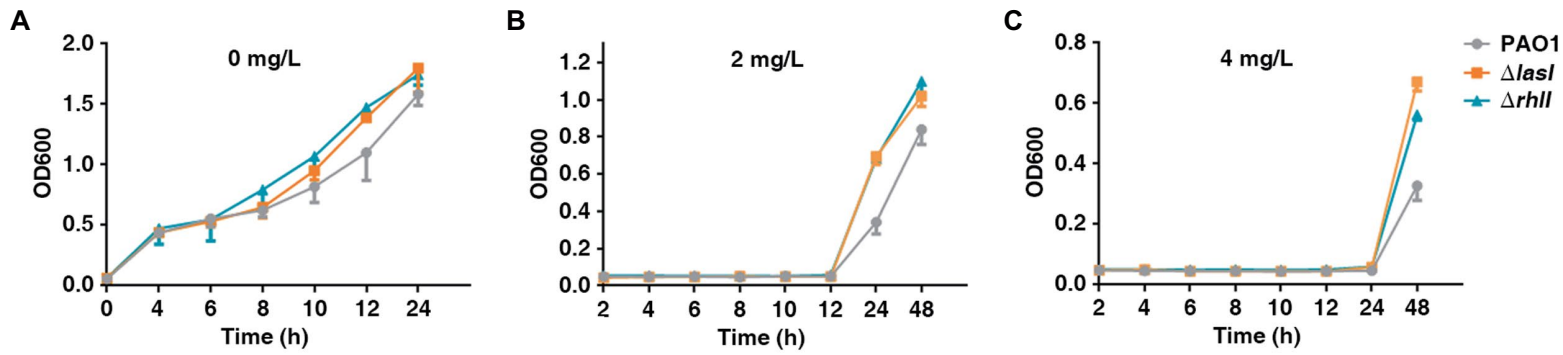
The growth rate of PAO1, PAO1Δ*lasI*, and PAO1Δ*rhlI* in the presence or absence of antibiotic selection. **(A–C)** PAO1, PAO1Δ*lasI*, and PAO1Δ*rhlI* were treated with 0, 2 or 4 mg/l of AMK.

The present study also investigated the growth rate of PAO1 wild type, PAO1Δ*lasI,* and PAO1Δ*rhlI* because the fitness cost was found to be significant in reversing HR. PAO1Δ*lasI* and PAO1Δ*rhlI* grew at a higher rate in the absence of antibiotics compared with PAO1 wide-type (Figure 5A). Further, PAO1Δ*lasI* and PAO1Δ*rhlI* also showed better relative growth rates upon exposure to AMK. Sub-MIC (2 or 4 mg/l) of AMK was used because only a few resistant subpopulations could grow under 2 or 4 mg/l of AMK. Results of this investigation showed that deficiency of *lasI* or *rhlI* favored the survival of PAO1 when exposed to 2 or 4 mg/l of AMK (Figures 5B,C). Moreover, previous transcriptome sequencing data (Pu et al., 2022) had found that deficiency of *lasI* or *rhlI* observably depressed expression of several genes, especially the virulence-related genes and impaired virulence such as pyocyanin, rhamnolipid synthesis and biofilm formation (Supplementary Table 3; Supplementary Figure 5). Therefore, it was inferred that *lasI* and *rhlI* deficiency-mediated downregulation of the genes resulted in lower consumption, which could be one of the strategies for bacteria to adapt to the Sub-MIC of antibiotic pressure before resistance is acquired.

## Discussion

Heteroresistance is a common resistance phenotype among clinical isolates that is the least investigated among the standard estimates of MICs. *P. aeruginosa* shows a high prevalence of HR, which affects the efficacy of clinical treatment courses based antibiotics (Band and Weiss, 2019). HR of *P. aeruginosa* to carbapenems, colistin, and cefepime has been previously demonstrated. However, there is need for a comprehensive study on the HR of *P. aeruginosa*. The current study aimed to first detect HR in *P. aeruginosa* to several antibiotics that are mainly used in clinical treatment. Results of the present study showed a high prevalence of *P. aeruginosa*-HR to carbapenems (MEM) and β-lactam (CAZ) as well as to quinolones (CIP) and aminoglycosides (AMK) antibiotics (Figure 1). To the best of our knowledge, the HR of *P. aeruginosa* to CIP, AMK, and CAZ shown in present study has not been previously reported. Therefore, we concluded that heteroresistance phenomenon may significantly contribute to the failure of treatment based on MEM, AMK, CIP, and CAZ antibiotics.

Recent studies have focused on the genetic mechanism-underlying heteroresistance. However, the present study identified QS system as a non-genetic mechanism for heteroresistance. In clinical isolated strains and hereditary stability model, HR was inversely associated with the level of LasI and RhlI (Figure 2A). In addition, gain-of-function and loss-of-function studies showed that QS repressed HR (Figure 3). Similarly, it has been found that indoles, which is a chemical signal produced by the resistant subpopulations of *E. coli*, can also help sensitive subpopulations adapt to antibiotics (Lee et al., 2010). In addition, resistant subpopulations of *Burkholderia cenocepacia* were shown to secrete putrescine and YceI proteins to improve the resistance of subpopulations that were sensitive to polymyxin B (Loutet et al., 2011). These studies show that chemical signals involved in the interaction between bacteria may be an important mechanism regulating HR.

The hallmarks of HR are the increase in the frequency of resistant subpopulations when exposed to antibiotic drugs and a decrease after removal of the antibiotic effect (Nicoloff et al., 2019). Previous studies have shown that increased resistance of the subpopulations resulted from spontaneous tandem amplifications, typically including known resistance genes. However, the procedure is costly and unstable, hence it may promote reversion to susceptibility in the absence of antibiotic selection (Nicoloff et al., 2019). In the current study, after 40 passages through antibiotic-free medium, the highly resistant subpopulations reverted to the heterogeneous resistant phenotype exhibited by the original population (Figure 2B). Furthermore, the course was accompanied by the change in expression of LasI, RhlI and QS-regulated virulence such as rhamnolipid, pyocyanin synthesis and biofilm formation (Supplementary Figures 2B–E). Accordingly, lower hamnolipid, pyocyanin synthesis and biofilm formation (Supplementary Figure 5), and better relative growth rates in strains deficient of *lasI* or *rhlI* upon exposure to antibiotics (Figure 5). In addition, the clinical isolates of *P. aeruginosa* naturally evolves *lasR* mutations during chronic infection. This forms the “cheater” populations that effectively stops the secretion of protease in support of utilizing public goods, thereby establishing a fitness advantage against antibiotics. Therefore, it was speculated that in the temporary presence of selective pressure, downregulation of QS genes may result in less fitness cost, which could be an important reason for the acquisition of heteroresistance against antibiotics.

OprD porin and efflux systems were introduced into mechanism research in the present study. Lower expression of *oprD* was observed in MEM and CAZ heteroresistant subpopulation compared with the dominant population (Figures 4B,D). While the genes associated with efflux increased in carbapenems, quinolones, and aminoglycosides heteroresistant subpopulation (Figures 4A,B,D). These results evidently confirmed the previous reports that OprD porin and efflux systems could have contributed to HR in *P. aeruginosa* on different antibiotics (Lin et al., 2019; Jia et al., 2020). Moreover, a little downregulation of *oprD* and obvious up-regulation of MexX/MexY was observed in PAO1 wild-types, PAO1Δ*lasI* and PAO1Δ*rhlI* treated with 2 mg/l of AMK (Figure 4E). These results could be related to the mechanism of *P. aeruginosa* resistance to antibiotics (Mesaros et al., 2007; Moradali et al., 2017; Xu et al., 2020). However, the expression of OprD and efflux systems showed no significant difference between wild-type and *lasI* or *rhlI* deficient strain (Figure 4E). The findings of the current study indicate that the inhibitory effect of QS system on the heteroresistance of *P. aeruginosa* is not mediated by *oprD* and efflux-associated genes.

In conclusion, the present study highlighted the regulatory role of the QS system in heteroresistance of *P. aeruginosa*, expanding the present understanding of the mechanism of antibiotic resistance in *P. aeruginosa*.

## Data availability statement

The original contributions presented in the study are included in the article/Supplementary material; further inquiries can be directed to the corresponding authors.

## Author contributions

YuL, YL, and CZ performed the experiments. YaL, YiL, QX, and DL described and discussed the results of the study. BH, CC, and YL were involved in data analysis and organization as well as preparation of the draft manuscript. All authors contributed to the article and approved the submitted version.

## Funding

This study was supported by grants from the National Natural Science Foundation of China (Grant No. 81871703), the Guangzhou Science Technology and Innovation Commission (Grant No. 202102010193), and Guangdong Provincial Hospital of Traditional Chinese Medicine (Grant numbers YN2018QJ01, YN2019QJ03, and YN2019MJ01).

## Conflict of interest

The authors declare that the research was conducted in the absence of any commercial or financial relationships that could be construed as a potential conflict of interest.

## Publisher’s note

All claims expressed in this article are solely those of the authors and do not necessarily represent those of their affiliated organizations, or those of the publisher, the editors and the reviewers. Any product that may be evaluated in this article, or claim that may be made by its manufacturer, is not guaranteed or endorsed by the publisher.

## Supplementary material

The Supplementary material for this article can be found online at: https://www.frontiersin.org/articles/10.3389/fmicb.2022.1017707/full#supplementary-material
