# Supplementary material for: Quorum sensing regulates heteroresistance in Pseudomonas aeruginosa
Source: Front Microbiol. 2022 Oct 28;13:1017707. doi: 10.3389/fmicb.2022.1017707 (PMC9650436; doi:10.3389/fmicb.2022.1017707)
Supplement: Supplementary file 1 [file Table_1.DOCX]

**Table S1. Bacterial strains and plasmids.**

| **Strains** | **Genotype or characteristics** | **Source** |
| --- | --- | --- |
| ***Pseudomonas aeruginosa*** | | |
| PAO1 | wild-type strain，Gm^S^，Amp^R^ | Our lab |
| PAO1 *ΔlasI* | deficiency of *lasI*，Gm^S^，Amp^R^ | Our lab ^[1]^ |
| PAO1 *ΔrhlI* | deficiency of *rhlI*，Gm^S^，Amp^R^ | Our lab |
| ***Plasmids*** | | |
| pROp200 | Control plasmid based on pBBR1 MCS-5，Gm^R^ | ^[2]^ |
| pROp200-*lasI* | pROp200 derivative，*P. aeruginosa* PAO1 *lasI* overexpression plasmid，controlled by the constitutive P*_tac_* promoter，Gm^R^ | Our lab^[3]^ |
| pROp200-*rhlI* | pROp200 derivative，*P. aeruginosa* PAO1 *rhlI* overexpression plasmid，controlled by the constitutive P*_tac_* promoter，Gm^R^ | Our lab^[3]^ |
| pROp200-*rhlR* | pROp200 derivative，*P. aeruginosa* PAO1 *rhlR* overexpression plasmid，controlled by the constitutive P*_tac_* promoter，Gm^R^ | Our lab^[3]^ |

Cm^R^, Gm^R^ and Amp^R^ stand for chloramphenicol, gentamycin and ampicillin resistance, respectively.

**References:**

[1] Zeng J, Zhang N, Huang B, et al. Mechanism of azithromycin inhibition of HSL synthesis in Pseudomonas aeruginosa[J]. Scientific reports, 2016, 6:24299.

[2] Lu P, Wang Y, Zhang Y, et al. RpoS-dependent sRNA RgsA regulates Fis and AcpP in Pseudomonas aeruginosa[J]. Molecular microbiology, 2016, 102 (2):244-259.

[3] Lu Y, Li H, Pu J, et al. Identification of a novel RhlI/R-PrrH-LasI/Phzc/PhzD signalling cascade and its implication in P. aeruginosa virulence[J]. Emerging microbes & infections, 2019, 8 (1):1658-1667.
