# Supplementary material for: Quorum sensing regulates heteroresistance in Pseudomonas aeruginosa
Source: Front Microbiol. 2022 Oct 28;13:1017707. doi: 10.3389/fmicb.2022.1017707 (PMC9650436; doi:10.3389/fmicb.2022.1017707)
Supplement: Supplementary file 2 [file Table_2.DOCX]

**Table S2. Sequences of RNA and DNA oligonucleotides**

| Number | Primers | Primer sequence^*^ (5’→3’) | Use for |
| --- | --- | --- | --- |
| **Primers for qPCR** | | | |
| **01** | lasI-F | CGTGCTCAAGTGTTCAAGGA | qRT-PCR |
| **02** | lasI-R | AAAACCTGGGCTTCAGGAGT |  |
| **03** | lasA-F | ACCAGATCCAGGTGAGCAAC | qRT-PCR |
| **04** | lasA-R | CGTTGTCGTAGTTGCTGGTG |  |
| **05** | rhlI-F | CTACCGGCATCAGGTCTTCA | qRT-PCR |
| **06** | rhlI-R | GTTTCGCTGCACAGGTAGG |  |
| **07** | rhlA-F | AGCTGGGACGAATACACCAC | qRT-PCR |
| **08** | rhlA-R | GACTCCAGGTCGAGGAAATG |  |
| **09** | mexA-F | GGCGACAACGCGGCGAAGG | qRT-PCR |
| **10** | mexA-R | CCTTCTGCTTGACGCCTTCCTGC |  |
| **11** | mexB-F | CAAGGGCGTCGGTGACTTCCAG | qRT-PCR |
| **12** | mexB-R | ACCTGGGAACCGTCGGGATTGA |  |
| **13** | mexE-F | TCATCCCACTTCTCCTGGCGCTACC | qRT-PCR |
| **14** | mexE-R | CGTCCCACTCGTTCAGCGGTTGTTCGATG |  |
| **15** | mexX-F | AATCGAGGGACACCCATGCACATCC | qRT-PCR |
| **16** | mexX-R | CCCAGCAGGAATAGGGCGACCAG |  |
| **17** | mexY-F | TAATGGTCCTTGGCCACCT | qRT-PCR |
| **18** | mexY-R | GCCCAACGACATCTACTTCAA |  |
| **19** | ampC-F | GGTGCAGAAGGACCAGGCACAGAT | qRT-PCR |
| **20** | ampC-R | CGATGCTCGGGTTGGAATAGAGGC |  |
| **21** | oprD-F | CGGCGACATCAGCAACACC | qRT-PCR |
| **22** | oprD-R | GGGCCGTTGAAGTCGGAGTA |  |
