# Supplementary material for: Quorum sensing regulates heteroresistance in Pseudomonas aeruginosa
Source: Front Microbiol. 2022 Oct 28;13:1017707. doi: 10.3389/fmicb.2022.1017707 (PMC9650436; doi:10.3389/fmicb.2022.1017707)
Supplement: Supplementary file 3 [file Table_3.DOC]

**Table S3. Differentially expressed genes (Top 15) in *lasI* deficient strains**

| **ID** | **Gene** | **PAOI** | ***ΔlasI*** | **logFC** | **PValue** |
| --- | --- | --- | --- | --- | --- |
| gene-PA3335 |  | 138 | 12 | 3.5 | 9.8E-121 |
| gene-PA3907 |  | 78 | 5 | 4.1 | 5.2E-114 |
| gene-PA3334 |  | 270 | 8 | 5.0 | 7.5E-113 |
| gene-PA4213 | phzD1 | 131 | 4 | 5.1 | 3.3E-103 |
| gene-PA1902 | phzD2 | 135 | 4 | 5.2 | 1.0E-102 |
| gene-PA3479 | rhlA | 198 | 8 | 4.7 | 2.2E-101 |
| gene-PA3329 |  | 126 | 6 | 4.4 | 5.5E-96 |
| gene-PA1432 | lasI | 1464 | 1 | 11.3 | 1.1E-95 |
| gene-PA1657 |  | 533 | 46 | 3.5 | 2.1E-94 |
| gene-PA4215 | phzF1 | 99 | 5 | 4.2 | 1.5E-93 |
| gene-PA4133 | ccoN | 616 | 20 | 4.9 | 1.9E-92 |
| gene-PA1904 | phzF2 | 103 | 6 | 4.2 | 7.3E-90 |
| gene-PA4128 |  | 138 | 12 | 3.5 | 1.7E-88 |
| gene-PA3905 |  | 78 | 5 | 4.1 | 7.5E-88 |
| gene-PA4217 |  | 270 | 8 | 5.0 | 1.4E-85 |

The calculation of Unigene expression uses RPKM method (Reads Per kb per Million reads)
