# Supplementary material for: Quorum sensing regulates heteroresistance in Pseudomonas aeruginosa
Source: Front Microbiol. 2022 Oct 28;13:1017707. doi: 10.3389/fmicb.2022.1017707 (PMC9650436; doi:10.3389/fmicb.2022.1017707)
Supplement: Supplementary file 4 [file Table_4.DOC]

**Table S4. Differentially expressed genes (Top 15) in *rhlI* deficient strains**

| **ID** | **Gene** | **PAOI** | ***ΔrhlI*** | **logFC** | **PValue** |
| --- | --- | --- | --- | --- | --- |
| gene-PA3476 | rhlI | 320 | 0 | 13.6 | 8.17E-165 |
| gene-PA3335 |  | 138 | 16 | 3.2 | 8.16E-75 |
| gene-PA4216 | phzG1 | 98 | 11 | 3.2 | 2.42E-61 |
| gene-PA1905 | phzG2 | 91 | 11 | 3.0 | 1.31E-53 |
| gene-PA1900 | phzB2 | 57 | 5 | 3.4 | 2.37E-52 |
| gene-PA4213 | phzD1 | 131 | 7 | 4.3 | 6.47E-42 |
| gene-PA3331 |  | 171 | 11 | 4.0 | 7.03E-41 |
| gene-PA1902 | phzD2 | 135 | 7 | 4.3 | 1.18E-39 |
| gene-PA3325 |  | 61 | 18 | 1.8 | 2.29E-38 |
| gene-PA4217 | phzS | 356 | 27 | 3.8 | 3.81E-37 |
| gene-PA4215 | phzF1 | 99 | 7 | 3.8 | 1.10E-36 |
| gene-PA1904 | phzF2 | 103 | 7 | 3.8 | 6.66E-35 |
| gene-PA3333 | fabH2 | 190 | 12 | 4.0 | 1.34E-34 |
| gene-PA4210 | phzA1 | 52 | 2 | 4.9 | 1.13E-32 |
| gene-PA3330 |  | 200 | 11 | 4.2 | 3.10E-31 |

The calculation of Unigene expression uses RPKM method (Reads Per kb per Million reads)
