# Supplementary material for: Quorum sensing regulates heteroresistance in Pseudomonas aeruginosa
Source: Front Microbiol. 2022 Oct 28;13:1017707. doi: 10.3389/fmicb.2022.1017707 (PMC9650436; doi:10.3389/fmicb.2022.1017707)
Supplement: Supplementary file 5 [file Data_Sheet_1.doc]

**SUPPLEMENTARY INFORMATION**

**Quorum sensing plays an important role in heteroresistance regulation of *Pseudomonas aeruginosa***

***Lu, Liu, et al***

**Supplementary Figure Legends**

Supplementary Figure 1. Heteroresistance of PAO1. Population analysis profiles (PAPs) were performed in five clones for each *P. aeruginosa* isolate. *P. aeruginosa* sensitive strain (ATCC 27853) and resistant strain (132A5) were used as control strain. AMK, amikacin; MEM, meropenem; CIP, ciprofloxacin; CAZ, ceftazidime.

Supplementary Figure 2. Expression of LasI and RhlI is negatively correlated with the HR of *P. aeruginosa*. (A) Biofilm formation potential in heteroresistant positive group of isolates was obviously lower than that in the negative group. (B) Expression of LasI and RhlI was down-regulated in the resistance of subpopulations as compared with original. Resistant subpopulations (Enrich) separated from PAPs test of original (ORI) was treated with the 2 mg/L of AMK. (C, D, E) Rhamnolipid, pyocyanin synthesis and biofilm formation was depressed under the treatment with antibiotics. PAO1 was treated with the indicated concentration of AMK.

Supplementary Figure 3. Stability of antibiotic resistance and heteroresistance. A resistant colony from the PAP test plates was re-streaked and grew on the same concentration of CIP (Enrich), followed by continuously passaging with or without antibiotic selection for 40 generations (1:1000 daily dilution). After serial passages in antibiotic-free medium, resistant subpopulations revert to the heterogeneous resistance phenotype displayed by the original population.

Supplementary Figure 4. Deficiency of *lasI* or *rhlI* in PAO promoted heteroresistance. PAPs was performed in PAO1 wild-type, PAO1Δ*lasI* and PAO1Δ*rhlI*.

Supplementary Figure 5. QS influences pyocyanin and rhamnolipid production, biofilm formation of *P. aeruginosa*. (A) Deficiency of *lasI* or *rhlI* inhibited pyocyanin production. PAO1 was cultured in LB for 24 h, and pyocyanin in the supernatant was measured. (B) Deficiency of *lasI* or *rhlI* inhibited rhamnolipid production. The indicated PAO1 strains were cultured in M9 minimal salts medium for 8 h at 37 °C, and rhamnolipid in the supernatant was measured. (C) Deficiency of *lasI* or *rhlI* inhibited biofilm formation. The indicated PAO1 strains were cultured in LB in the 12-well plates for 24 h at 37°C. The biofilm was quantified by measuring solubilized crystal violet staining biofilm cells at OD600. *, *P*< 0.05; **, *P*< 0.01; ***, *P*< 0.001.
